# Supplementary material for: Impact of Voluntary, Community and Social Enterprise (VCSE) Organisations Working with Underserved Communities with Type 2 Diabetes Mellitus in England
Source: Healthcare (Basel). 2023 Sep 8;11(18):2499. doi: 10.3390/healthcare11182499 (PMC10530582; doi:10.3390/healthcare11182499)
Supplement: Supplementary file 1 [file healthcare-11-02499-s001.zip › healthcare-2471715-supplementary.pdf]

SUPPLEMENTARY MATERIALS

Supplementary Material S1: VAS Diabetes topic guide for interviews

Introduction: Thank you for attending for today's interview. This research programme is to understand what makes community and voluntary organisations work for local communities and individuals living with diabetes, what we can learn from your experiences and what can be done better or differently. I expect that this interview will last 30-45 minutes and we will discuss 10 or so questions which we feel are important.

If you're ready, I will start recording our conversation now.

1) How did you find out that you had diabetes?
    - Did you visit your GP?
    - Through hospital admission?
    - Self-recognition of the symptoms?
    - Family member/friends?

2) Please could you tell me about your diabetes journey from finding out you had it through to your current management?
    - What have you been invited to? Did you attend everything or were there barriers?
    - Who have you seen for diagnosis/treatment? Including indirect services e.g. weight management or mental health
    - Which services have you preferred and why? Are they culturally/financially/resource appropriate?
    - Do the services feel well connected?

3) What does having diabetes mean to you?
    - Does it feel like a manageable/treatable condition?
    - Does it worry you?
    - Do you know other people who have diabetes?
    - Do you feel it impacts your overall wellbeing and/or mental health?

4) What do you do to help manage your diabetes?
    - Do you know what support is available for people living with diabetes? Is it easy to find? Were you signposted to appropriate care by professionals or others?
    - Where do you access care? Who supports you e.g. CVS/GP/diabetes clinic/family/friends
    - Do you take medication? What type of medication? Do you take or use any traditional or home remedies or therapies?
    - Do you think any other aspect of your wellbeing is affected (e.g. mental health)?
    - Do you use any apps or medical devices to help support you? Are they useful?

5) Where do you get your information from about diabetes?
    - What is most useful to you?

- Is there a special/specific diet that you follow? Or any particular foods or ingredients that you include or definitely can't eat?
- Are there any changes that you have made to your exercise or physical activity levels?

6) Do you think that the advice and information that you have been given was appropriate for you?
    - Did it feel manageable/achievable?
    - Culturally/financially/resource appropriate?

7) Did you see your GP/doctor for your diabetes? [If no, Why Not? Then move on to Q 8]
   Thinking about the visits to your GP/doctors surgery for your diabetes:
    - Why did you access this service?
    - What did they do well?
    - What could they do differently?
    - What should they do more of?

8) Did you visit your hospital/ specialist diabetes clinic for your diabetes? [If no, Why Not?? Then move on to Q 9]
   Thinking about the visits to your hospital/ specialist diabetes clinic for your diabetes:
    - Why did you access this service?
    - What did they do well?
    - What could they do differently?
    - What should they do more of?

9) Did you visit a CVS provider [insert name of organisation] since being diagnosed with diabetes? [If no, Why Not?? Then move on Q 10]
   Thinking about the visits to your CVS provider [insert name of organisation]:
    - Why did you access this service?
    - What did they do well?
    - What could they do differently?
    - What should they do more of?

10) If you were designing a service for your community to help manage diabetes, what do you think it should look like?
    - Where would it be located?
    - Who would work there?
    - How often would you want to use it? Would you prefer face-to-face, online, group vs 1:1, formal (e.g. clinic) vs informal (e.g. diabetes café)?
    - What information or service would you want them to provide?

Supplementary Material S2: COREQ checklist

COREQ (COnsolidated criteria for REporting Qualitative research) Checklist

A checklist of items that should be included in reports of qualitative research. You must report the page number in your manuscript where you consider each of the items listed in this checklist. If you have not included this information, either revise your manuscript accordingly before submitting or note N/A.

Topic	Item No.	Guide Questions/Description	Reported on Page No.
**Domain 1: Research team and reflexivity**			
Personal characteristics			
Interviewer/facilitator	1	Which author/s conducted the interview or focus group?	6
Credentials	2	What were the researcher's credentials? E.g. PhD, MD	6
Occupation	3	What was their occupation at the time of the study?	1
Gender	4	Was the researcher male or female?	1
Experience and training	5	What experience or training did the researcher have?	6
Relationship with participants			
Relationship established	6	Was a relationship established prior to study commencement?	7
Participant knowledge of the interviewer	7	What did the participants know about the researcher? e.g. personal goals, reasons for doing the research	7
Interviewer characteristics	8	What characteristics were reported about the inter viewer/facilitator? e.g. Bias, assumptions, reasons and interests in the research topic	6
**Domain 2: Study design**			
Theoretical framework			
Methodological orientation and Theory	9	What methodological orientation was stated to underpin the study? e.g.<br>grounded theory, discourse analysis, ethnography, phenomenology, content analysis	5
Participant selection			
Sampling	10	How were participants selected? e.g. purposive, convenience, consecutive, snowball	5-7
Method of approach	11	How were participants approached? e.g. face-to-face, telephone, mail, email	7
Sample size	12	How many participants were in the study?	8
Non-participation	13	How many people refused to participate or dropped out? Reasons?	n/a
Setting			
Setting of data collection	14	Where was the data collected? e.g. home, clinic, workplace	7

Presence of nonparticipants	15	Was anyone else present besides the participants and researchers?	n/a
Description of sample	16	What are the important characteristics of the sample? e.g. demographic data, date	8-9
Data collection			
Interview guide	17	Were questions, prompts, guides provided by the authors? Was it pilot tested?	6-7
Repeat interviews	18	Were repeat inter views carried out? If yes, how many?	n/a
Audio/visual recording	19	Did the research use audio or visual recording to collect the data?	7
Field notes	20	Were field notes made during and/or after the inter view or focus group?	7
Duration	21	What was the duration of the inter views or focus group?	7
Data saturation	22	Was data saturation discussed?	n/a
Transcripts returned	23	Were transcripts returned to participants for comment and/or correction?	7
**Domain 3: analysis and findings**			
Data analysis			
Number of data coders	24	How many data coders coded the data?	7
Description of the coding tree	25	Did authors provide a description of the coding tree?	
Derivation of themes	26	Were themes identified in advance or derived from the data?	7
Software	27	What software, if applicable, was used to manage the data?	n/a
Participant checking	28	Did participants provide feedback on the findings?	7
Reporting			
Quotations presented	29	Were participant quotations presented to illustrate the themes/findings?<br>Was each quotation identified? e.g. participant number	10-16
Data and findings consistent	30	Was there consistency between the data presented and the findings?	17
Clarity of major themes	31	Were major themes clearly presented in the findings?	10-16
Clarity of minor themes	32	Is there a description of diverse cases or discussion of minor themes?	17

Developed from: Tong A, Sainsbury P, Craig J. Consolidated criteria for reporting qualitative research (COREQ): a 32-item checklist for interviews and focus groups. *International Journal for Quality in Health Care*. 2007. Volume 19, Number 6: pp. 349 – 357

Once you have completed this checklist, please save a copy and upload it as part of your submission. DO NOT include this checklist as part of the main manuscript document. It must be uploaded as a separate file.
